# The Diabetes Management Education Program in South Texas: An Economic and Clinical Impact Analysis

**DOI:** 10.3389/fpubh.2017.00345

**Published:** 2017-12-18

**Authors:** Bita A. Kash, Szu-Hsuan Lin, Juha Baek, Robert L. Ohsfeldt

**Affiliations:** ^1^School of Public Health, Texas A&M University, College Station, TX, United States

**Keywords:** diabetes management education program, economic impact, healthcare cost savings, clinical benefits, South Texas counties, cost differentials model, UKPDS risk engine model

## Abstract

**Introduction:**

Diabetes is a major chronic disease that can lead to serious health problems and high healthcare costs without appropriate disease management and treatment. In the United States, the number of people diagnosed with diabetes and the cost for diabetes treatment has dramatically increased over time. To improve patients’ self-management skills and clinical outcomes, diabetes management education (DME) programs have been developed and operated in various regions.

**Objective:**

This community case study explores and calculates the economic and clinical impacts of expanding a model DME program into 26 counties located in South Texas.

**Methods:**

The study sample includes 355 patients with type 2 diabetes and a follow-up hemoglobin A1c level measurement among 1,275 individuals who participated in the DME program between September 2012 and August 2013. We used the Gilmer’s cost differentials model and the United Kingdom Prospective Diabetes Study (UKPDS) Risk Engine methodology to predict 3-year healthcare cost savings and 10-year clinical benefits of implementing a DME program in the selected 26 Texas counties.

**Results:**

Changes in estimated 3-year cost and the estimated treatment effect were based on baseline hemoglobin A1c level. An average 3-year reduction in medical treatment costs per program participant was $2,033 (in 2016 dollars). The total healthcare cost savings for the 26 targeted counties increases as the program participation rate increases. The total projected cost saving ranges from $12 million with 5% participation rate to $185 million with 75% participation rate. A 10-year outlook on additional clinical benefits associated with the implementation and expansion of the DME program at 60% participation is estimated to result in approximately 4,838 avoided coronary heart disease cases and another 392 cases of avoided strokes.

**Conclusion:**

The implementation of this model DME program in the selected 26 counties would contribute to substantial healthcare cost savings and clinical benefits. Organizations that provide DME services may benefit from reduction in medical treatment costs and improvement in clinical outcomes for populations with diabetes.

## Introduction

Diabetes is a major chronic health conditions in the United States (U.S.), and it is also one of the top 10 leading cause of death in the U.S. (1). Without proper treatment, diabetes can lead to serious health conditions. Diabetes affects people of all ages; current estimates suggest that 29.1 million people, or 9.3% of the total U.S. population, have either type 1 or type 2 diabetes, and one-fourth of them do not know that they have diabetes (1). Among those diagnosed with diabetes, about 90–95% have type 2 diabetes (2).

## Background and Rationale

While diabetes affects people of all ages, previous studies have observed differences in diabetes prevalence among specific race/ethnicity groups (2, 3). Non-Hispanic Blacks and Hispanic have higher prevalence of diabetes compared to non-Hispanic whites (2), while the Hispanic population has the highest prevalence of type 2 diabetes when compared to all other race/ethnicity groups (4). Studies show the incidence and prevalence of diabetes are increasing rapidly worldwide (5, 6). If the diabetes epidemic is not properly addressed by the medical and public health professions and the U.S. healthcare policy, it is estimated that one in every three people in the U.S. could have diabetes by 2050 (1, 7).

Over one-third of the total U.S. adults population have pre-diabetes, which lead to high risk for developing type 2 diabetes, but only 10% of people recognizes it (3). People with diabetes have a higher risk of developing other serious health problems and chronic diseases. Without proper treatment, lifestyle changes, and monitoring of condition, diabetes complications can be severely disabling and even life-threatening. Some potential health complications associated with diabetes include cardiovascular disease (CVD), nerve damage (neuropathy), kidney damage (nephropathy), and eye damage (retinopathy) (8, 9). Over 70% of patients with diabetes die of cardiovascular-related causes (10, 11). The risk of heart disease for people with diabetes is two to four times higher than those without diabetes (12). Kidney failure results primarily from complications associated with diabetes, and it accounts for about 44% of all new cases of kidney failure, or nearly 50,000 people, in 2011 (13). Most people living with kidney failure require long-term dialysis treatment and often have to undergo kidney transplants, which can add to the overall cost of treatment associated with diabetes.

The cost of diabetes to the U.S. healthcare system is enormous. In 2012, the estimated total cost for diabetes treatment, including both direct and indirect costs, was $245 billion, which has increased rapidly from $98 billion in 1997 and $174 billion in 2007 (12, 14). With an estimated projection that one in every three adults in the U.S. will be diagnosed with diabetes by 2050 (7), the total healthcare-related costs for diabetes are expected to increase even further (15). In particular, people diagnosed with diabetes have been reported to spend an average of 2.3 times greater in annual medical expenditures compare to those without diabetes (12). Today, for every five dollar spent on health care, approximately one dollar is toward patient with diabetes (7).

To improve the early detection, monitoring, self-management, and quality of life of people with diabetes, a variety of interventions, such as patient education programs and comprehensive disease management programs, have been developed and tested in the U.S. and beyond. Effective education can lead to effective disease management and opportunities to avoid future medical treatments and development of comorbidities (16). Diabetes management education (DME) assists people with diabetes or at risk for developing diabetes to understand the nature of their medical condition and learn how to modify behaviors to control the progression of diabetes and other associated diseases. Evidence-based research in the area of DME interventions have shown that these programs can potentially improve patients’ self-management skills and clinical outcomes (10, 17). Some DME programs have provided effective patients education for diabetes care, prevention of short- and long-term complication, and enhancement of healthcare outcomes (18–20).

In this study, the research team worked with a model DME center (“the Center”) located in a city that serves 20 surrounding counties in South Texas in 2014. The Center is one of the key community-based healthcare institutions in the area. It is affiliated with several health systems and an academic health center and university, and offers a DME program to support diabetic patients (participants in the DME program) living in surrounding counties. The Center’s DME programs help patients with diagnosed diabetes to self-manage their diabetes and teach those with pre-diabetes or those who are at risk of developing diabetes to prevent or delay the onset of the disease. The program offers diabetes self-management classes in both English and Spanish by certified diabetes educators, registered nurses, and nutritionists. The 8-h classes teach fundamental information about diabetes, the importance of healthy diet and physical activity, as well as how to test blood sugar levels. The program participants receive complimentary, follow-up labs, and one-on-one consultation. To improve the health and wellness of the people and increase the impact of the DME program in this geographic service area, the Center proposed to expand its service area from 20 surrounding counties to 26 counties in 2014. This community case study is to explore the economic and clinical impact of expanding the existing model of DME program to 26 selected counties around the Center.

Considering the high cost of diabetes for patients, health systems, and the U.S. healthcare system, this paper sheds some light on how effective one model DME program can help increase people’s knowledge about diabetes and diabetes self-management, and ultimately in saving healthcare costs long term as participation in the program increases. Using the results of the Center’s DME program as a model, which is already in place and tracks program cost and participant biomarkers, the research team extrapolated the impact of expanding this model of DME program to a larger services area and achieving higher market penetration with this expanded geographic target area.

## Methods

### Sample

To examine the impact of expanding the Center’s DME program model to 26 counties on saving healthcare-related cost and reducing future diabetes-related “bad events,” such as coronary heart disease (CHD) and stroke, we used the Center’s DME program de-identified participant-level data for the analysis. The data included a total of 1,275 individual who participated in the DME program between September 2012 and August 2013. We included only participants with a diagnosis of type 2 diabetes and those who had a follow-up hemoglobin A1c level measurement, and excluded those with gestational or type 1 diabetes diagnosis and those with only one initial hemoglobin A1c measurement. As a result, the final sample size included a total of 355 observations. This study was approved by the Texas A&M University’s Institutional Review Board (IRB).

### DME Program Expansion: Estimated 3-Year Cost Savings

We used Gilmer and colleagues’ (21) approach to predict healthcare costs associated with type 2 diabetes. Gilmer and colleagues (21) used 3 years of patient records and medical claims to assess the impact of patient baseline hemoglobin A1c level, presence of CVD, and depression on subsequent healthcare cost among adults with diabetes (21). In the study, the total costs were calculated as the sum of costs from the claim or encounter data from the first hemoglobin A1c measurement until disenrollment, death, or end of study period. To calculate a 3-year total cost estimate for each individual in the study sample, each individual’s observed that cumulative cost was divided by the number of days enrolled since the first day of hemoglobin A1 measurement until disenrollment, death, or end of study period. This average daily cost estimate for each individual was multiplied by 1,095.75 (i.e., the number of days in 3 years, 3 × 365.25) to generate a standardized 3-year cost estimate (21). Detailed description of the methodology is described in Gilmer et al.

To project the estimated change in 3-year treatment costs, we used the abovementioned Gilmer model’s results to generate estimates of program treatment effects in terms of estimated changes from baseline hemoglobin A1c level at 12 months, stratified by baseline hemoglobin A1c level. The Gilmer model’s results (summarized in Gilmer’s Table 3, last row) report the estimated change in 3-year medical care costs associated with a unit change in hemoglobin A1c levels from baseline hemoglobin A1c levels of 10, 9, 8, and 7%. We used a regression model to extrapolate the estimated costs savings for individuals with baseline hemoglobin A1c levels greater than 10%. A quadratic model was preferred to a linear extrapolation model based on goodness of fit.

Taking into account the average cost per participant in the Center’s DME program ($650; information provided by the Center), we also estimated the cost savings for the Center’s DME program expansion into 26 counties, based on various levels of the program participation rates (5, 10, 15, 20, 50, and 75%).

### DME Program Expansion: Estimated 10-Year Clinical Benefit

We used the estimated 12-month program treatment effect for hemoglobin A1c level and systolic blood pressure to produce pre- and post-program values to use in the U.K. Prospective Diabetes Study (UKPDS) Risk Engine Excel file. The UKPDS Risk Engine is a type 2 diabetes-specific risk calculator that uses 20 years of UK Prospective Diabetes Study data to estimate future risks of developing CHD and stroke (22). The UKPDS is widely used to project changes in clinical risk factors, such as hemoglobin A1c and blood pressure, on clinical outcome, such as stoke and CVD (23). We used the sample median of self-reported years with diabetes from the sample of the Center’s DME program participants for the analysis. For the other clinical indicators included in the risk engine calculator, such as total cholesterol and high-density lipoprotein-cholesterol, we used the estimated baseline values reported in the Jacobs et al. (24), assuming no pre/post change in these factors due to program participation. Appendix 1 in Suplementary Material Data Sheet 1 shows the complete set of clinical values used in the UKPDS Risk Engine Model.

Estimates of event risks for the pre- and post-program values were generated using the UKPDS Risk Engine for specific patient segments, delineated by age (age<45, assumed median age 31; age 45–64, assumed median age 55; and age 65+, assumed median age 75), gender (male or female), race (black or not-black), and smoking status (current smoker or not). The estimated changes in event risks for each of the patient segments were then applied to the size of the estimated population with diabetes for each patient segment and summed over patient segments to calculate the estimated impact of disease event rates.

## Results

### Service Area Expansion Goal: from 20 to 26 Counties

A total of 355 patients with type 2 diabetes were included in the analysis. Table 1 presents the sociodemographic and health characteristics of the sample participants.

**Table 1:** Participants’ sociodemographic and health characteristics (*n* = 355).

**Gender**		**Education level**	
Female	54%	Less than 12 years	20%
Male	46%	12 years	47%
**Age group**		13 to <16 years	18%
Less than 30	2%	16+ years	15%
30–44	13%	**Type of insurance coverage**	
45–54	26%	County indigent program	38%
55–64	36%	Private insurance	42%
65+	23%	Medicare	11%
**Race and ethnicity**		Medicaid	2%
Hispanic	68%	Others	0.5%
Non-Hispanic White	28%	No insurance	6.5%
Non-Hispanic Black	3%	**% Participants by class type**	
Other	1%	English 8 h	79%
**Language spoken**		English 4-part 2 h	16%
English	96%	Spanish 8 h	5%
Spanish	4%	**Initial hemoglobin A1c level**	
**Difficulty understand English**		6.5–<8%	44%
Yes	4%	8–<10%	29%
No	96%	10–<12%	14%
**Family history of diabetes**		Greater than 12%	13%
Yes	84%		
No	16%		

About 54% of the Center’s DME program participants with type 2 diabetes were female and the majority of the participants were Hispanic, followed by non-Hispanic White, non-Hispanic Black, and other race. Most of the participants were between age 45 and older, with few people under the age of 30. About 80% of the sample participants had at least 12 years of education, and almost all the participants speak English, with only 4% of participants have difficulty understanding English. When choosing a type of DME class to attend, the majority of the participants choose English version of the class, and only 5% of participants attended the Spanish DME courses.

When looking at the type of insurance coverage patients had, the majority of the patients had some kind of insurance coverage. Many participants were either enrolled in the county indigent program (38%) or had private insurance (42%). The remaining 13.5% of participants had Medicare, Medicaid, or other types of health insurance. About 6.5% of the participants did not have health insurance at all.

Patients included in the analysis were previously diagnosed with type 2 diabetes. With the majority of patients with initial hemoglobin A1C level between 6.5 and 10%, about 25% of patients started the program with initial hemoglobin A1c level equal or greater than 10%.

### Estimated 3-Year Cost Savings Result

The results of Gilmer and colleagues’ study (21) in predicting healthcare costs associated with type 2 diabetes indicate that cost savings are greater for a unit change from higher baseline hemoglobin A1c levels, with no significant improvement in costs associated with a reduction in hemoglobin A1c level from 7 to 6%. Therefore, a sample selection of participants with relatively high initial A1c levels will produce more significant cost savings when enrolled in a DME program such as the Center’s program.

The summary results reported in Gilmer et al. (21) reflect only reported costs estimates for unit reductions in hemoglobin A1c levels from a baseline level of hemoglobin A1c equal to 10% or lower. We extrapolated the 3-year treatment cost saving for hemoglobin A1c level greater than 10% from a regression model (see Appendix 2 and 3 in Supplementary Material Data Sheet 1). Table 2 shows the projections of 3-year treatment cost savings for the Center’s DME program participants with type 2 diabetes, stratified by the baseline hemoglobin A1c level.

**Table 2:** Projections of 3-year treatment cost savings for The Center’s program participants with type 2 diabetes, by baseline hemoglobin A1c level.

Baseline hemoglobin A1c level	Sample share (%)	Δ 3-year Cost (-1 unit A1c)	Est. 12 m Δ A1c	Est. 3-year Δ Cost	Weighted share
Greater than 11%	16.0	$1,080^a^	3.84	$4,956	$793.03
10–11%	10.5	$1,404^a^	2.27	$3,129	$328.49
9–10%	14.1	$1,374	1.07	$1,467	$206.91
8–9%	13.5	$1,303	0.99	$1,283	$173.27
7–8%	24.2	$373	0.25	$93	$22.39
Less than 7%	21.7	$0	0.00	0	$0.00
Overall Avg cost savings ($2002)					$1,524
*Inflation adjustment ($2002–$2016)*					1.342
Overall Avg cost savings ($2016)					$2,033

*^a^Extrapolated using predicted treatment costs savings from regression model in Appendix 3 in Supplementary Material Data Sheet 1*.

The proportion of the Center’s DME program participants in each baseline hemoglobin A1c level category is shown in column 2 of Table 2. The estimated 3-year cost changes associated with a unit decrease in hemoglobin A1c level reported in Gilmer et al. (21) (extrapolated for baseline hemoglobin A1c level greater than 10% as previously noted) for different baseline hemoglobin A1c levels are reported in column 3. Treatment effect estimates for each baseline hemoglobin A1c level cohort are reported in column 4. Note that the hemoglobin A1c level treatment effects were zero for program participants with a baseline hemoglobin A1c level less than 7%, but substantial changes were recorded in the baseline hemoglobin A1c levels greater than 10%.

Table 2 summarizes the estimated change in 3-year medical care costs associated with a one unit change in hemoglobin A1c from the Gilmer model (with extrapolation to higher levels of baseline hemoglobin A1c as noted above). However, the estimated total treatment effect for program participants with baseline hemoglobin A1c levels greater than 11% was the 3.84 unit reduction in hemoglobin A1c level. Thus, simply using the cost savings for a one unit reduction in hemoglobin A1c for this group would likely result in a substantial underestimate of the actual reduction in 3-year costs attributable to the program, given the 3.84 unit decrease in hemoglobin A1c observed in the Center’s data.

To project the cost impact of the 3.84 unit reduction in hemoglobin A1c level, we assumed that the cost savings associated with one unit hemoglobin A1c reductions shown in Appendix 2 in Supplementary Material in Data Sheet 1 were additive. Specifically, we estimated the cost impact of a total 3.84 unit reduction in A1c for participants with baseline A1c greater than 11% as the sum of the cost savings of a unit reduction from a hemoglobin A1c level greater than 11 to 11% ($1,080), plus the cost impact of another unit reduction from a hemoglobin A1c level of 11 to 10% ($1,404)—a cumulative change 2 units of A1c, plus the cost impact of another unit reduction from a hemoglobin A1c level of 10 to 9% ($1,374)—a cumulative change of 3 units of hemoglobin A1c, plus the cost impact of a final 0.84 reduction in hemoglobin A1c level (a cumulative change of 3.84 units), estimated as $1,095 (0.84 × $1,303, the estimated change in costs for a unit reduction in A1c from a hemoglobin A1c level of 9 to 8%). A similar approach was used to project the cost impact of the 2.3 unit reduction in hemoglobin A1c level for program participants in the baseline hemoglobin A1c level of 10–11% cohort, the 1.1 unit reduction for the hemoglobin A1c level of 9–10% cohort, and the 1.0 unit reduction for the hemoglobin A1c level of 8–9% cohort.

The overall 3-year treatment cost savings were estimated as the weighted average of the cost savings within each baseline hemoglobin A1c level strata, weighted by the sample share of program participants in each strata, as shown in Table 2. As the Gilmer model reported treatment cost estimates in 2002-equivalent dollars, the 2002-dollar costs estimate was inflated to 2016-equivalent dollars using the U.S. Bureau of Labor Statistics’ Consumer Price Index calculator (25). The resulting estimate is an average 3-year reduction in medical treatment costs of $2,033 per program participant.

Using the 2011 information from CDC about the type 2 diabetes prevalence by gender, race, and age groups, and the U.S. Census Bureau estimated population in 2012 for each of the 26 target counties, we extrapolated that a total of 187,303 people in the 26 target counties had type 2 diabetes in 2012. We then estimated the total healthcare cost savings after subtracting the total program cost calculated at $650 per participant from the calculated cost differential at the six different levels of program participation rates for the target counties. Table 3 shows the total healthcare cost savings for the 26 target counties increases as the participation rate increases: when 5% of the people diagnosed with diabetes participate in the DME program, the estimated cost saving would be about $12 million. At 20% participation rate, the estimated total cost savings would reach almost $50 million for the 26 target counties. With a participation rate of 75% (over 140,000 people diagnosed with diabetes participating in the expanded program), the estimated total healthcare cost savings for the 26 targeted counties would be over $185 million.

**Table 3:** Proposed DME program in 26 targeted counties: estimated healthcare cost saving over 3 years.

Program participation rate (%)	Projected number of participants	Total DME program costs	Total medical care cost savings	Medical cost savings net of program cost in 26 counties
5	9,365	$6,087,250	$18,486,510	$12,399,459
10	18,730	$12,174,500	$36,973,020	$24,798,917
15	28,095	$18,261,750	$55,459,530	$37,198,376
20	37,461	$24,349,650	$73,948,014	$49,597,834
50	93,652	$60,873,800	$184,869,048	$123,994,586
75	140,477	$91,310,050	$277,301,598	$185,991,879

### Estimated 10-Year Clinical Benefit Result

The UKPDS Risk Engine provides risk estimate for non-fatal and fatal CHD and non-fatal and fatal stroke. Using the UKPDS, we are able to estimate the number of events avoided over a 10-year period for CHD and stroke. An estimated 187,303 people diagnosed with type 2 diabetes reside in the 26 target counties. Table 4 shows the estimated number of “bad events” avoided over a 10-year period among people with diabetes in the 26 targeted counties. Assuming 60% of people in the 26 target counties participated at the Center’s DME program and were able to better manage their diabetes, we can expect to observe 4,838 fewer CHD and 392 fewer stroke events over a 10-year period among diabetic patients in the 26 target counties (26 fewer CHD cases and 2 fewer stroke cases per thousand diabetes patients). We summed the CHD and stroke cases to provide estimates for the number of avoided CVD events over a 10-year period. At 60% participation rate, it is estimated that 5,630 CVD events and 268 fatal CVD events were avoided during this time period. The number of CVD and fatal CVD event avoided as reported in Table 4 is likely to be underestimated because CVD usually includes angina, myocardial infarction, stroke, and peripheral vascular disease (26). As the percent participation rate increases, we can expect to see an increase in the number of adverse events avoided.

**Table 4:** Estimated number of events avoided over a 10-year period among people with diabetes in 26 South Texas Counties^a^.

	Percent participation rate				
	100% *N*	90% *N*	80% *N*	70% *N*	60% *N*
**Cardiovascular disease (CVD)^b^**					
Total number of CVD avoided	8,716	7,845	6,973	6,101	5,230
Total number of fatal CVD avoided	446	402	357	312	268
**Coronary heart disease (CHD)**					
Total number of CHD avoided	8,063	7,257	6,450	5,644	4,838
Total number of fatal CHD avoided	445	400	356	311	267
**Stroke**					
Total number of stroke avoided	653	588	523	457	392
Total number of fatal stroke avoided	2	1	1	1	1

*^a^Total population with diabetes in 26 Texas target counties: 187,303*.

*^b^Cardiovascular disease defined as CHD events and stoke events; does not include other types of CVD events*.

## Discussion

Based on the analysis of the participant-level data provided by the Center, we concluded that investment in community-based diabetes education self-management programs can provide effective education that helps increase patients competency in managing their diabetes and potentially reducing medical cost and avoid preventable adverse health outcomes. We estimate that the expansion of a DME Program, such as the program at the Center into the 26 target counties in South Texas will likely result in total healthcare cost savings of $50 million over 3 years, assuming a 20% participation rate among people with type 2 diabetes in the 26 counties. Therefore, it is important to engage in community-level recruitment of participants into these types of diabetes education and management programs. Furthermore, a 10-year outlook on additional clinical benefits associated with the implementation and expansion of the DME program (at 60% participation) could result in about 4,838 cases of avoided CHD and another 392 cases of avoided strokes in the 26 county areas.

However, it is possible that at a low participation rate, the patients participating consist of those best suited for the program, whereas at high participation rates, patients less suited to the program also will be participating. Thus, the magnitude of the program treatment effect may diminish as the participation rate increases. The results in Table 3 assume that the treatment effect is constant across participation levels, which might cause an upward bias in cost savings estimates at the highest participation rates.

Conversely, as the participation rate of the DME program increases, it is likely that the Center will observe economies of scale resulting in reduced fixed costs associated with the DME program operations. This reduction in program cost can be due to managerial economies of scale, such as specialization of the workforce, better operational management, investment in human resource, and other factors affecting reductions in fixed cost. The assumed absence of economies of scale in program costs should at least partially offset the impact of the potential bias in estimated diabetes costs savings on net program cost estimates.

### Lessons Learned/Recommendations

Diabetes is currently one of the most common chronic diseases in the U.S. Without proper education and management, it will be able to increase patients’ risks for numerous serious health problems, which also increase the healthcare costs for the patient and the community. This case study demonstrated that organizations that provide DME programs, such as the program discussed in the case, have the potential to save medical treatment costs and also improve clinical outcomes.

This study is not without limitation. First, the study included only data and results from a 1-year short-term DME program with only patients who were diagnosed with type 2 diabetes. Second, we also assumed that the last A1c level observation of each patient in the data is constant over the projected time interval. Third, we took a conservative approach when analyzed the Center’s data and extrapolated cost and clinical benefit impacts for the 26 target counties. Due to the conservative methodological approach used in this analysis to extrapolate the Gilmer model’s results to the higher baseline A1c levels observed in the data, coupled with an assumption of no economies of scale in DME program fixed costs, the projected cost savings associated with the DME program may be underestimated. Fourth, this DME program includes education and training to teach and help patients better manage and control their diabetes. We are unable to separate the parts of the DME program and study their impact on A1c levels, although it is possible that parts of the DME program could be more effective than the others. In this paper, we studied this DME program as a whole in helping patients manage their diabetes.

The positive results of this pilot study helped the Center to expand their services area. Future studies are needed to include larger sample size and longitudinal data to examine the effectiveness of this DME program in helping patients avoid preventable adverse health outcome. There is also potential for comparative effectiveness studies as educational programs are implemented at various sites using different technologies and skill levels.

## Author Contributions

BK, SHL, and RO contributed to the conception and design of the work, data acquisition, and analysis. BK, SHL, JB, and RO drafted, reviewed, and approved the manuscript.

## Conflict of Interest Statement

The authors declare that the research was conducted in the absence of any commercial or financial relationships that could be construed as a potential conflict of interest.
