# Supplementary material for: The Diabetes Management Education Program in South Texas: An Economic and Clinical Impact Analysis
Source: Front Public Health. 2017 Dec 18;5:345. doi: 10.3389/fpubh.2017.00345 (PMC5741603; doi:10.3389/fpubh.2017.00345)
Supplement: Supplementary file 1 [file Data_Sheet_1.docx]

| **Appendix 1: Summary of Clinical Values Used in the UKPDS Risk Engine Model** | | |
| --- | --- | --- |
|  | Pre | Post |
| A1c | 8.9 | 7.7 |
| SBP | 135 | 131 |
| TotC^†^ | 210 | -- |
| HDL^†^ | 41 | -- |
| Years with Diabetes Mellitus | 2 | -- |
| Atrial Fib | No | -- |

**APPENDIX**

^†^Estimated baseline values reported in Jacobs et al. (24).

| **Appendix 2. Estimated Three-Year Treatment Cost Savings Associated with a Unit Reduction in A1c Level, by Baseline Hemoglobin A1c Level** | | | | | |
| --- | --- | --- | --- | --- | --- |
| Baseline Hemoglobin A1c level (%) | | | | | |
| **>11 (to 11)** | **11 (to 10)** | **10 (to 9)** | **9 (to 8)** | **8 (to 7)** | **7 (to 6)** |
| $1,080* | $1,404* | $1,374 | $1,303 | $373 | $0 |

Source: Gilmer et al. (21).
*Results extrapolated using regression model by authors.

| **Appendix3 : Regression Model Used for Extrapolation of 3-year Change in Treatment Costs Associated with Unit Change in Hemoglobin A1c Level to Higher Baseline Hemoglobin A1c Level Categories** | | | | | | | | | | |
| --- | --- | --- | --- | --- | --- | --- | --- | --- | --- | --- |
| *Regression Statistics* | | | | |  | | | | | |
| Multiple R | | | 0.975121061 | |  | | |  | | |
| R Square | | | 0.950861083 | |  | | | | | |
| Adjusted R Square | | | 0.852583249 | |  | | | | | |
| Standard Error | | | 262.2110453 | |  | | | | | |
| Observations | | | 4 | |  | | | | | |
|  | | | | | | | | | | |
| ANOVA | | | | | | | | | | |
|  | *df* | | *SS* | *MS* | | | *F* | | | *Significance F* |
| Regression | | 2 | 1330434.37 | 665217.1839 | | | 9.6752 | | 0.2217 | |
| Residual | | 1 | 68754.63 | 68754.6323 | | |  | |  | |
| Residual | | 3 | 1399189.00 |  | | |  | |  | |
|  | | |  | |  |  | | | | |

|  | *Coefficients* | Standard Error | t Stat | p-value |
| --- | --- | --- | --- | --- |
| Intercept | -13467.871 | 10493.69 | -1.2834 | 0.42138 |
| Baseline A1c | 3005.1652 | 2613.40 | 1.1499 | 0.45568 |
| (Baseline A1c)^2 | -151.31745 | 160.4569 | -0.9430 | 0.51866 |
